# The MAPK Pathway-Based Drug Therapeutic Targets in Pituitary Adenomas

**DOI:** 10.3389/fendo.2019.00330

**Published:** 2019-05-22

**Authors:** Miaolong Lu, Ya Wang, Xianquan Zhan

**Affiliations:** ^1^Key Laboratory of Cancer Proteomics of Chinese Ministry of Health, Xiangya Hospital, Central South University, Changsha, China; ^2^Hunan Engineering Laboratory for Structural Biology and Drug Design, Xiangya Hospital, Central South University, Changsha, China; ^3^State Local Joint Engineering Laboratory for Anticancer Drugs, Xiangya Hospital, Central South University, Changsha, China; ^4^National Clinical Research Center for Geriatric Disorders, Xiangya Hospital, Central South University, Changsha, China

**Keywords:** MAPK, ERK, p38, JNK, signaling pathway, pituitary tumor, biomarker, therapeutic drug

## Abstract

Mitogen-activated protein kinases (MAPKs) include ERK, p38, and JNK MAPK subfamilies, which are crucial regulators of cellular physiology, cell pathology, and many diseases including cancers. For the MAPK signaling system in pituitary adenomas (PAs), the activation of ERK signaling is generally thought to promote cell proliferation and growth; whereas the activations of p38 and JNK signaling are generally thought to promote cell apoptosis. The role of MAPK in treatment of PAs is demonstrated through the effects of currently used medications such as somatostatin analogs such as SOM230 and OCT, dopamine agonists such as cabergoline and bromocriptine, and retinoic acid which inhibit the MAPK pathway. Further, there are potential novel therapies based on putative molecular targets of the MAPK pathway, including 18beta-glycyrrhetinic acid (GA), dopamine-somatostatin chimeric compound (BIM-23A760), ursolic acid (UA), fulvestrant, Raf kinase inhibitory protein (RKIP), epidermal growth factor pathway substrate number 8 (Eps8), transmembrane protein with EGF-like and two follistatin-like domains (TMEFF2), cold inducible RNA-binding protein (CIRP), miR-16, and mammaliansterile-20-like kinase (MST4). The combined use of ERK inhibitor (e.g., SOM230, OCT, or dopamine) plus p38 activator (e.g., cabergoline, bromocriptine, and fulvestrant) and/or JNK activator (e.g., UA), or the development of single drug (e.g., BIM-23A760) to target both ERK and p38 or JNK pathways, might produce better anti-tumor effects on PAs. This article reviews the advances in understanding the role of MAPK signaling in pituitary tumorigenesis, and the MAPK pathway-based potential therapeutic drugs for PAs.

## Introduction

Pituitary adenomas (PAs) are commonly benign tumors, accounting for about ten percent of intracranial tumors (1, 2), and are clinically divided into functioning PAs (FPAs) and non-functioning PAs (NFPAs) (3, 4). It can cause significant morbidity and mortality (5). The molecular mechanisms in tumorigenesis and functional regulation of PAs have been extensively studied. This review article focuses on the roles of mitogen-activated protein kinase (MAPK) in PA tumorigenesis and the MAPK pathway-based potential therapeutic targets for PAs. MAPKs mainly include three subfamilies based on the conserved Thr-Xaa-Tyr motif signature: ERK1/2, p38, and JNK (Jun N-terminal kinase) (6), which are activated by multiple factors such as growth factors and stress. The activation of ERK promotes cell proliferation; whereas, the activations of p38 and JNK promote cell apoptosis. Studies demonstrate that MAPKs are involved in multiple cellular processes, such as cell differentiation, proliferation, apoptosis, inflammation, stress responses, and immune defense (7–9).

**Figure 1:**
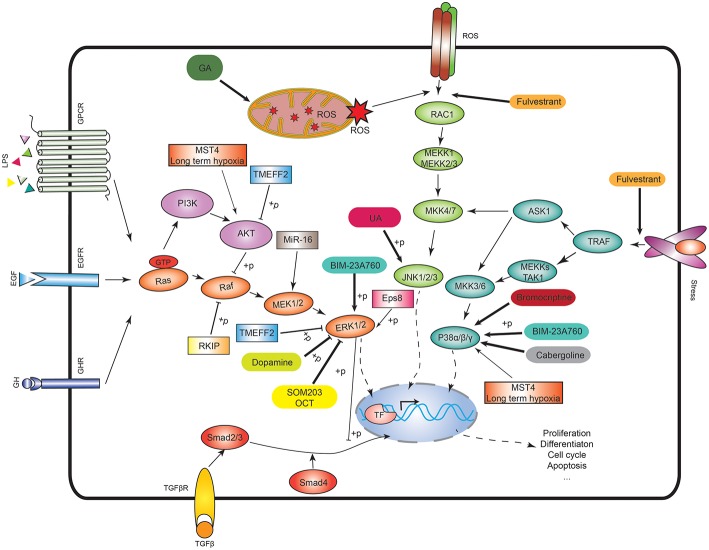
MAPK signaling pathways and the potential therapeutic targets. In the ERK signaling, Ras activates the serine/threonine protein kinase Raf to activate MEK1/2, then MEK1/2 phosphorylates the ERK1/2. In p38 signaling, TNF receptor-associated factor (TRAF) activates ASK1, TAK1, or MEKK1, which activates MKK3/6, and then MKK3/6 phosphorylates p38 isoforms. In JNK signaling, RAC1 activates MEKK1 or MEKK2/3 to activate MKK4/7, and then MKK4/7 phosphorylates JNK1/2/3. The ASK1 in the p38 signaling also activates MKK4/7 to crosstalk with JNK signaling. ROS means reactive oxygen species. GA means 18beta-glycyrrhetinic acid. BIM-23A760 is a dopamine–somatostatin chimeric compound. OCT means octreotide. SOM230 and OCT are somatostatin analogs. Rectangle means the potential drug targets.

The MAPK signaling pathways play important roles in cell dissemination, survival, and drug resistance of human cancers including PAs (2, 10–12). With the in-depth studies of the MAPK signaling pathway network, MAPK pathways-based target-specific drugs have been developed, and some drugs has been used for clinical trials; and the relevance of MAPK in response and resistance to antitumor drugs has also been recognized (Figure 1 and Table 1). Because of the important roles of MAPK signaling pathways in tumorigenesis, the use of the MAPK signaling pathways as therapeutic targets has continuously been considered as a promising strategy for cancer therapy. This review highlights new advances in the role of MAPK signaling in pituitary tumorigenesis and development, the key molecules in this pathway network, and anti-pituitary tumor drugs targeting MAPK signaling pathway.

**Table 1:** Drugs or molecules involved in MAPK signaling pathway in PAs.

**Drugs or molecules involved in MAPK signaling pathway**			**Tumor subtype**	**Molecular mechanism**	**Biological effect**	**Research models**	**References**
Therapeutic drugs	Somatostatin analogs (SSTs)	SOM230 (FDA approved)	GH-secreting PAs	SOM230 combines with somatostatin receptor subtype 2 (SSTR2) and inhibits ERK pathway	Inhibits GH release and proliferation of tumor cells	Primary GH-secreting adenoma cells, and rat pituitary cell line (GH3)	(13–15)
			PRL-secreting PAs	SOM230 combines with SSTR5 and inhibits ERK pathway	Inhibits PRL release and proliferation of tumor cells	Primary PRL-secreting adenoma cells, and rat pituitary cell line (GH3)	(13–15)
			Corticotropin-secreting PAs	SOM230 binds to SSTR5 not SSTR2 to inhibit ERK pathway	Suppresses CRH-induced ACTH release and decreases urinary free cortisol (UFC), and serum cortisol	AtT20/D16V mouse tumor cells, and pituitary dependent Cushing's disease patients	(16–18)
			Gonadotroph PAs	Suppresses the phosphorylation of ERK	Reduces GnRH-induced LH secretion	Mouse gonadotroph LβT2 cells	(19)
		Octreotide (OCT) (FDA approved)	GH-secreting PAs	OCT combines with SSTR2 and inhibits ERK pathway	Inhibits GH-release and proliferation of tumor cells	Primary GH-secreting adenoma cells, and rat pituitary cell line (GH3)	(13–15)
			Gonadotroph PAs	Suppresses the phosphorylation of ERK	Reduces GnRH-induced LH secretion	Mouse gonadotroph LβT2 cells	(19)
	Dopamine and Dopamine agonists	Dopamine	PRL-secreting PAs	Suppresses the phosphorylation of ERK1/2	Reduces PRL secretion	PRL-secreting cells (GH4ZR7), and primary pituitary cells	(20)
		Bromocriptine (BRC) (FDA approved)	PRL secreting pituitary adenoma	Activates p38 MAPK	Increases GH3 cell apoptosis	Rat pituitary cell line (GH3)	(21)
		Cabergoline (CAB) (FDA approved)	PRL secreting pituitary adenoma	Activates p38 MAPK	Increases GH3 cell apoptosis	PRL-D2S cells	(22)
	TGFβ		Lactosomatotroph PAs	Cross-talks with ERK pathway	Decreases the cell proliferation	Rat lactosomatotroph pituitary adenoma cells (GH3B6)	(23)
	18beta-glycyrrhetinic acid (GA)		GH-secreting PAs	Activates mitochondria-mediated ROS, JNK and P38 pathways	G0/G1 phase arrest and increases apoptosis rate	Rat pituitary adenoma-derived MMQ and GH3 cells	(24)
			PRL-secreting PAs				
	The dopamine–somatostatin chimeric compound	BIM-23A760	NFPAs	Activates ERK1/2 and p38 pathways	Inhibits cell proliferation and induces apoptosis	Primary NFPA cells	(25)
	Ursolic Acid (UA)		ACTH-secreting PAs	Upregulates JNK phosphorylation and increases the degradation of Bcl-2	Induces apoptosis of AtT20 cells and decreases ACTH secretion	AtT20 pituitary corticotroph cell line	(26)
	Fulvestrant		GH-secreting PAs	Activates PTEN/MAPK signaling pathways	Increases apoptotic cell death	Rat pituitary cell line (GH3)	(27)
			PRL-secreting PAs				
Potential targets	Raf kinase inhibitory protein (RKIP)		Somatotroph PAs	Non-phosphorylated RKIP binds to and inhibits Raf1 kinase and attenuates MAPK signaling	Low levels of RKIP correlate to poor clinical response to SSTs	Patients with active acromegaly	(28)
	Epidermal growth factor pathway substrate number 8 (Eps8)		Gonadotroph PAs	Increases phosphorylated ERK	Promotes proliferation and cell survival	Mouse gonadotroph LβT2 cells	(29, 30)
	TMEFF2 (transmembrane protein with EGF-like and two follistatin-likedomains)		Corticotroph PAs	Inhibits phosphorylation of AKT and ERK1/2	Decreases proliferation of corticotrope cells and reduces secretion of ACTH	AtT20 pituitary corticotrope tumor cells GH3 pituitary lactosomatotroph tumor cells	(31)
	Cold inducible RNA-binding protein (CIRP)		Corticotroph PAs	Induces cyclinD1 and decreases p27 expression via Erk1/2 signaling pathway	Promotes cells proliferation and tumor recurrence	AtT20 pituitary corticotroph cell line	(32)
	MiR-16		Non-functional PAs	Inhibits ERK/MAPK pathway activity via the suppression of MEK1 expression	Inhibits cell proliferation and induces apoptosis and cell-cycle arrest	Human HP75 tumor cells	(33)
	Mammaliansterile-20-like kinase (MST4)		Gonadotroph PAs	Activates p38 MAPK and AKT during long-term hypoxia	Increases colony formation and accelerates cell proliferation	Mouse gonadotroph LβT2 cells	(34)

*PAs, pituitary adenomas. NFPAs, Non-functional pituitary adenomas*.

## The ERK Pathway in PAs

The ERK/MAPK pathway delivers signals from cellular surface receptors via ERK pathway. Briefly, different cellular surface receptors such as EGFR, GPCR, and RKT are activated by the corresponding extracellular factors (e.g., growth factors, hormones, and stresses) to activate Ras and small GTPase. The activated Ras-small GTPase complex recruits Raf kinase to the cell membrane and activates it. Then, Raf activates MEK (MAPK and ERK kinase) through phosphorylation. The phosphorylated MEK subsequently activates ERK through phosphorylation (35) (Figure 1). Rafs include Raf-1, A-Raf, and B-Raf. Raf-1 can bind to the pro-apoptotic kinases, such as mammalian sterile-twenty-like-2 (MST2) and apoptosis signal-regulating kinase (ASK1), to involve in cell apoptosis (36). Raf-1 also exerts scaffolding function in regulation of the Rho pathway (37). Typically, cytokines and growth factors binding to TKR activate ERK1/2, which transduces the signals into its upstream Ras/Raf/MEK pathway. In PAs, H-Ras mutations have been identified in two cases of prolactinomas, which indicates that Ras/ERK takes part in regulation of PAs (38, 39). Overexpression of B-Raf is pre-dominantly observed in NFPAs (40). The downstream kinases of B-Raf in ERK MAPK pathway are also over-activated in NFPAs, growth hormone (GH)-secreting PAs, ACTH-secreting PAs, and prolactinomas. The phosphorylation levels at pSer217/221 of MEK 1/2 and pThr183 of ERK1/2 are significantly increased in these PAs compared to controls (41), which indicates that Raf/MEK/ERK pathway acts as a pro-proliferative role in PAs.

### The Effects of ERK MAPK on Different-Origin PAs

The effect of ERK signaling on PAs depends on the PA subtypes. (i) In lactotroph cells, the ERK signaling exerts different effect on cell proliferation based on the exposure time. Short-time activation of the ERK (24–96 h) leads to increased proliferation in rat pituitary lactotroph or somatolactotroph cell lines *in vitro* (42, 43). However, long-time activation of ERK (over 6 days) promotes somatolactotroph cell differentiation into a lactotroph cell phenotype, and then decreases proliferation and tumorigenicity with time (44). Thus, persistent activation of ERK signaling produces anti-proliferative and anti-tumorigenic effects in somatolactotroph cells. (ii) In somatotroph cells, ERK signaling produces pro-proliferative effects. Protein kinase A (PKA) and C (PKC) pathways regulate ERK signaling. PKA pathway activates ERK signaling, and leads to improved proliferation in GH-secreting cells. PKC stimulates ERK signaling and increases cell proliferation through regulating GH-releasing hormone (GHRH) (45). ERK pathway is necessary for somatotrophs to produce GH. In somatotroph PAs, GH-releasing hormone (GHRH) can promote cell proliferation through activating ERK signaling (46). In addition to regulation of cell proliferation, ERK signaling also contributes to GH secretion by somatotrophs (47). Somatostatin (SST) analogs are used in clinical treatment of GH-secreting PAs due to its anti-proliferative effect on somatotroph cells. SST treatment results in a reduction of pERK1/2 expression and a significant increase in p27 protein expression. In addition, cell proliferation is driven by cell cycle which is regulated by a series of cyclins and cyclin dependent kinases (CDKs). A cyclin-dependent kinase (CDK) inhibitor has a negative effect on cell-cycle progression which has a synergistic effect with SST analogs (13). (iii) In gonadotroph cells, gonadotropin-releasing hormone (GnRH) can activate ERK, p38, and JNK signaling in the LβT2 gonadotroph cell lines to contribute to production of luteinizing hormone (LH), and GnRH phosphorylated ERK via PKC-dependent pathways (48). Most of NFPAs originate from gonadotroph cells where B-Raf is upregulated and ERK is over-activated relative to control pituitary tissues (40, 49). (iv) In thyrotroph cells, ERK cascade has anti-proliferative effects. The ERK pathway is activated to cause growth arrest after thyrotroph adenomas are treated with thyroid hormone (50). And (v) In corticotroph cells, ERK signaling is activated to produce pro-proliferative effects (51).

### ERK MAPK Pathway-Targeted Pharmacological Treatments of PAs

#### Somatostatin (SST) Analogs Treatment

SST inhibits cell growth, through G protein-coupled receptors to inhibit the release of growth factors and angiogenesis, and increases apoptosis. The majority of NFPAs express SST receptors on cell membranes. An appropriate concentration of SST analogs (octreotide or SOM230) can inhibit the release of GH, prolactin (PRL), and their α-subunit in GH-secreting PAs, PRL-secreting PAs, ACTH-secreting PAs, and NFPAs, respectively (14–18). Their anti-tumor effects are in that SST analogs can inactivate ERK signaling pathways; for example, octreotide acts on both ERK and PI3K/Akt signaling pathways, and SOM230 acts on ERK signaling pathway (13, 52). Octreotide can bind to and activate SST receptor subtype-2 (SSTR2) and SSTR5, while pasireotide (SOM230) can activate SSTR1, 2, 3, and 5 (53, 54). A study shows that octreotide or SOM230 reduces cell proliferation and pERK1/2 expression in rat somatotroph cell line GH3 (13). Octreotide also blocks the transient G0/G1 cell cycle to produce a cytostatic effect on GH3 cell proliferation (55). SST analogs (octreotide and pasireotide) also decrease secretion of LH induced by GnRH in LβT2 cells (19), and inhibit NFPA cell viability *in vitro* (56–59). The SST analogs are the primary medical therapy to treat acromegaly for maintenance of GH homeostasis and shrinkage of tumor size (60–62). Moreover, octreotide and lanreotide bind with high affinity to SSTR2, and with low affinity to SSTR3 and SSTR5. The decreased expression of SSTR2 in tumor is associated with lack of response to SST analogs (63, 64). Some studies demonstrate that SST analogs exert their anti-proliferative effects on somatotroph cells through inhibition of ERK signaling (13).

#### Dopamine and Dopamine Agonists Treatment

Hypothalamic dopamine suppresses the production of pituitary PRL (65). Dopamine acts via the D2 receptor to inhibit cAMP/PKA and MAPK signaling pathways to control PRL-secretion and lactotroph proliferation (20). Dopamine agonists such as bromocriptine (BRC) and cabergoline (CAB) are primary medical therapy drugs for prolactinomas and idiopathic hyperprolactinemia and prolactinomas (66). Dopamine agonists target the dopamine D2 receptor (D2R) subtype to exert its anti-tumor effects. D2R-activated ERK signaling cascades inhibit the synthesis and release of PRL in the pituitary. D2R includes D2L and D2S isoforms. Overexpression of D2L elevates PRL, and overexpression of D2S reduces PRL. The ratio of D2L to D2S affects PRL-secretion of lactotroph cells (67). A study shows that when pituitary tumor cells are treated with dopamine, D2S activation stimulates ERK signaling to inhibit lactotroph cell proliferation (68).

#### TGFβ Treatment

TGFβ is widely considered to be a tumor suppressor (69). However, TGFβ1 produces weak growth inhibitory effect on pituitary tumor cells. TGFβ mainly uses Smad signaling pathway to convey signals from cytosol to nucleus to regulate expression of genes that control cell cycle progression (70). In addition, TGFβ can also use non-Smad signaling pathway to convey signals, such as MAPK pathway (71). TGFβ1 treatment decreases pituitary tumor cell proliferation, and this inhibitory effect is amplified by MEK inhibitors, because TGFβ1/Smad pathway cross-talks with MEK/ERK1/2 pathway (23). It clearly demonstrates that inhibition of MEK/ERK1/2 pathway synergizes with TGFβ1 to inhibit pituitary tumor cell proliferation.

### Potential Targets Related to ERK MAPK Signaling Pathway for PA Treatment

Components and regulators of the ERK MAPK pathway are all potential targets for treating pituitary tumors. (i) Raf kinase inhibitory protein (RKIP). RKIP is a modulator of MAPK signaling, which inhibits Raf-1 phosphorylation to inhibit Ras/Raf-1/MEK/ERK signaling pathway (72–74). RKIP interferes with Raf-1 in several mechanisms. One is that Raf-1 binding to RKIP causes the conformational change of Raf-1 (75). Another is that RKIP inhibits phosphorylation of MAPK-MEK-1 to interfere with the interaction between two kinases (76). Moreover, protein kinase C (PKC) can negatively regulate the roles of RKIP because PKC phosphorylates RKIP to cause the separation of RKIP from Raf-1 (73). Studies demonstrate that the low expression level of RKIP in GH-secreting adenomas is correlated with less GH and IGF-1 reduction with SST analog therapy because RKIP can inhibit the phosphorylation of Raf1 kinase to attenuate the activity of MAPK signaling pathway (28).

(ii) Epidermal growth factor pathway substrate number 8 (Eps8). Over-expression of Eps8 and over-activation of Raf, MEK, and ERK in the ERK signaling pathway promote cell proliferation and survival in PAs (41). Also, Eps8 is a substrate of receptor tyrosine kinases (RTKs) in the ERK signaling pathway (Figure 1), which can enhance EGF-dependent mitogenic signaling (29). Eps8 expression is significantly higher in human gonadotroph adenomas relative to controls. Upregulation of EGFR protein and phosphorylation of ERK are demonstrated in Eps8-overexpressing LβT2 cells. EGF ligand stimulation leads to increased proliferation in Eps8-overexpressing LβT2 cells. MAPK kinase inhibitor (PD98059) can abrogate the proliferative effects. Silence of Eps8 also inhibits cell proliferation, which suggests that Eps8 promotes pituitary tumor cell proliferation through enhancing the Raf/MEK/ERK signaling (30). Therefore, Eps8 is a potential drug target for PA treatment.

(iii) Retinoic acid (RA). RA has antiproliferative effect in corticotroph cell, and long-term treatment with RA has some clinical efficacy in patients with Cushing's disease (77). The mechanism of RA anti-tumor effects is shown with the expression of TMEFF2 (transmembrane protein with EGF-like and two follistatin-likedomains) that inhibits phosphorylation of AKT and ERK1/2 (31). TMEFF2 is significantly downregulated in corticotropinomas relative to control tissues, which suggests that TMEFF2 might be a tumor suppressor. Silence of TMEFF2 in pituitary corticotroph cell line AtT20 promotes cell proliferation, while over-expression of TMEFF2 inhibits cell proliferation (31). Thus, TMEFF2 is a potential therapeutic target for ACTH-secreting adenomas.

(iv) Cold inducible RNA binding protein (CIRP). The underlying mechanism of cold-shock protein (CIRP) in its role in tumorigenesis is through its induction of cyclinD1 which decreases p27 expression via ERK1/2 signaling (32, 78). CIRP is significantly upregulated at the mRNA and protein levels in multiple cancers (79, 80), including human corticotroph adenomas relative to normal pituitary tissues. CIRP over-expression is associated with recurrence of corticotroph adenomas in murine models (32). CIRP-overexpressing AtT20 cells have increased cell proliferative abilities. Tumor xenografts generated by CIRP-overexpressed AtT20 cells are significantly larger than AtT20 cells with normal level of CIRP expression.

## The p38 Pathway in PAs

The p38 MAPK includes isoforms p38α, p38β, p38γ, and p38δ, with ~60% of sequence similarity among four isoforms (81). Of these, p38α (MAPK14) and p38β (MAPK11) are highly expressed in various tissues, p38γ (MAPK12/ERK6) in muscle, and p38δ (MAPK13/SAPK4) in lung and kidney (34, 82). The p38 MAPK plays vital roles in cell responses to stimulators, including proinflammatory cytokines and environmental stresses such as ultraviolet irradiation and heat shock; and is involved in cellular differentiation, cell migration, and inflammation. Activation of p38 kinases are due to phosphorylations at Thr180 and Tyr182 within Thr-Gly-Try motif (83). This canonical phosphorylation is regulated by MKK3 and MKK6, which are highly selective for p38 MAPKs (84–86) (Figure 1). There exist a large body of substrates of p38 MAPKs both in cytoplasm and nucleus, such as transcription factors (p53, MEF2, CHOP, and ATF2), and other protein kinases (MNK1/MNK2, and MSK1/MSK2) which in turn phosphorylate other important proteins (Hsp27, and eIF-4E) (81). In PAs, p38 MAPK plays an important role in immune escape. Tumor immune escape means that tumor cells escape from the body's immune system recognition and attacking to survive and proliferate in the body. When tumor cells appear in the healthy body, the body's immune surveillance system can recognize and specifically remove these “non-self” tumor cells through natural and acquired immunity to prevent the development of tumors (87). However, in some cases, malignant cells can escape the immune surveillance of the body through various mechanisms to rapidly proliferate and form tumors. Studies demonstrate that phosphorylated p38 stimulates the expression of matrix metalloproteinase 9 (MMP9), which is involved in accelerating the process of tumor immune escape (88). In addition, studies based on murine gonadotroph cells LβT2 reveal that mammalian sterile-20-like kinase (MST4) is upregulated in the levels of mRNA and protein to promote cell proliferation by activating p38 MAPK and AKT during long-term hypoxia (89).

### PA Treatment Related to p38 MAPK Signaling

Previous studies demonstrate that p38 MAPK is associated with apoptosis, and drugs that activate this pathway can thereby induce apoptosis in pituitary tumor cells. A study found that dopamine agonists such as BRC and CAB activate p38 pathway to induce cell apoptosis; for example, when BRC is used to treat rat lactosomatotroph GH3 cells, BRC activates p38 MAPK and promotes cell apoptosis. Moreover, p38 MAPK inhibitors (SB202190, SB203580) completely inhibit BRC-induced p38 MAPK activation and cell apoptosis (21). Similarly, CAB also activates p38 MAPK and induces apoptosis in PRL-D2S cells (22). In addition to dopamine agonists, the natural compound 18beta-glycyrrhetinic acid (GA) extracted from liquorice can induce several types of tumor cell apoptosis (24, 90). In rat PA cells MMQ and GH3, GA can induce cellular damage, decrease cell viability, and cause G0/G1 phase arrest to contribute to cell apoptosis (91). GA can enhance the phosphorylation of JNK and p38, and these effects are abrogated through pretreatment with JNK inhibitor (SP60125) or p38 inhibitor (SB203580). Furthermore, the fact that ROS inhibitor (NAC) abolished the activation of JNK and P38 suggests that GA exerts the anti-PA effects by activating ROS/MAPKs (JNK and P38)-dependent pathway (91). BIM-23A760, a dopamine-somatostatin chimeric compound, by activating p38 and ERK1/2, inhibits cell proliferation and demonstrates cytotoxic effects in primary culture of NFPAs (25).

### Potential Targets Related to p38 MAPK Signaling Pathway for PA Treatment

In recent years, with the development of next-generation sequencing (NGS), a large number of non-coding RNAs have been identified (92, 93). Non-coding RNAs not only deepen our understanding of tumorigenesis and development, but also provide new directions for the diagnosis and treatment of tumors. Studies demonstrate that microRNA-16 (miR-16) is significantly downregulated in PAs compared to the healthy controls, and overexpression of miR-16 reduces the protein expressions of phosphorylated p38, VEGFR2, MMP-9, and NF-kB in HP75 cells, which suggests that miR-16 is involved in PA cell proliferation and angiogenesis via VEGFR2/p38/NF-κB pathway (94). Studies show that miR-16 suppresses MEK1 expressions thereby inhibiting ERK/MAPK pathway activity, leading to inhibition of cell proliferation, cell-cycle arrest, and apoptosis in PAs (33). Therefore, miR-16, as a regulator of p38 MAPK, might be a diagnostic biomarker and a target of PAs. In addition, based on miR-6-related studies, miR-6-protein and/or miR-6-lncRNA interactions are also worth further exploring for discovery of potential therapeutic targets. Further, MST4 promotes cell proliferation by activating p38 MAPK under long-term hypoxia. Therefore, MST4 might be a target for PA treatment (34).

## The JNK MAPK Pathway in PAs

The JNK MAPK pathway is mainly activated by various stress stimuli, including oxidative stress, UV irradiation, osmotic shock, heat shock, and proinflammatory cytokines (95), and plays vital roles in controlling proliferation, cell growth, apoptosis, inflammatory, and immune responses (96–98). JNK includes three isoforms: JNK1 and JNK2 are extensively distributed in different tissues, and JNK3 is mainly expressed in testis, heart, and brain (99, 100). JNK is activated by a cascade reaction: stress signals are delivered by small GTPases (Rac, Rho, and cdc42) to a series of kinase cascades, and eventually MKK4/7 activates JNKs (101). In addition, MKK4/7 can also be activated by a member of the germinal center kinase (GCK) family to activate JNKs (95). Moreover, MKK4 might also activate p38 MAPKs (p38α and p38δ), which lets JNK pathway cross-talk with p38 MAPK pathway (34). The activated JNKs are translocated from cytoplasm to nucleus where it can regulate the activity of multiple transcription factors (ATF-2, Elk-1, Smad4, p53, NFAT4, and Stat3) (102).

JNK pathway has been reported to be involved in many kinds of cancers, including retinoblastoma, melanoma, colorectal cancer, breast cancer, and ovarian cancer; and these cancers exhibit the elevated JNK activities (103–107). While there are limited studies on the role of the JNK pathway in the initiation and progression of pituitary tumors, several studies have demonstrated that alteration of JNK in the pituitary gland could be associated with pituitary tumoregensis. For instance, mice with a conditionally inactivated JNK1 in nestin-expressed cells (JNK1^Δ*NES*^) are used to study the effects of JNK1 signaling on glucose metabolism. Unexpectedly, the decreased somatic growth and increased thyroid axis activities are observed in JNK1^Δ*NES*^ mice with decreased levels of circulating GH and IGF1 (108). Another study shows that ablation of Jnk genes in anterior pituitary gland of mice leads to increased energy expenditure, and decreased obesity compared to control mice; and pituitary thyroid-stimulating hormone (TSH) and blood thyroid hormone (T4) are increased (109). Thus, JNK signaling might be involved in pituitary tumorigenesis (110).

### JNK MAPK Pathway-Targeted Pharmacological Treatments of PAs

The first one is ursolic acid (UA), a triterpenoid compound found in food, medical herbs, and other plants (111), which has antitumor effects in a number of tumors such as hepatocellular carcinoma, melanoma, breast cancer, colorectal cancer, bladder cancer, and prostate cancer (112–117). In the treatment of PAs, UA decreases cell viability and induces apoptosis in AtT20 cells by upregulating JNK phosphorylation. JNK signaling can also cross-talk with UA-induced mitochondrial apoptotic signaling transduction through phosphorylation and degradation of Bcl-2 (26). Moreover, GA, as described above, can induce cellular cytotoxicity and apoptosis by enhancing the phosphorylation of JNK. With further research progress, it is strongly believed that more drugs will be developed to target JNK MAPK pathway.

## The MAPK Pathway Network in PAs

ERK, p38, and JNK signaling pathways both independently and concordantly contribute to pituitary tumorigenesis. Thus, some chemotherapeutic drugs for PAs may target several subfamilies of the MAPK signaling pathway at the same time. For example, fulvestrant is an estrogen receptor antagonist without agonist effects (118), which is approved in the EU and USA to treat post-menopausal women who have hormone-sensitive advanced breast cancer, after prior antiestrogen therapy (119). In the treatment of PAs, recent studies reveal that fulvestrant significantly suppresses the cell viability and invasion of rat PA GH3 cells by simultaneous regulation of ERK1/2, JNK1/2, and p38 signaling pathways (27). GA exerts anti-tumor effects against PAs through enhancing the activations of JNK and p38 MAPK signaling pathways (91). BIM-23A76 that is a dopamine-somatostatin chimeric compound demonstrates the function of inhibiting cell proliferative and cytotoxic effects by activating p38 and ERK1/2 (25).

The comprehensive pathway-network analysis of multiple sets of proteomic data in PAs (120–124) reveals that MAPK signaling abnormalities, including ERK-MAPK signaling pathway, are significantly associated with PAs (125), and that some important molecules such as ERK, p38, JNK, Ras, Akt, NF-kB, TNF, and TGFb1 in MAPK signaling pathway network are identified in human PAs. In addition, the regulatory effect of MAPK cascades on cell differentiation, proliferation, survival and apoptosis interact with other transduction pathways (126). For instance, both PI3K-Akt and Raf/MEK/ERK pathways synergistically promote cell proliferation at the initial stage of PAs (127). Another study demonstrates the critical role of ERK1/2 and cAMP in determination of tumoural phenotype in PAs (128). Thus, a combination of drugs that target pathways which cross-talk with MAPK signaling may produce a more effective treatment for PAs.

In the MAPK network system in PAs, the activation of ERK is generally thought to promote cell proliferation and growth; whereas the activations of p38 and JNK are generally thought to promote cell apoptosis. The MAPK signaling pathway can be targeted by several mechanisms. Two types of combination strategies can be used: (i) A single drug to target ERK pathway and p38 or JNK pathway. For example, BIM-23A76 inhibits cell proliferation through targeting ERK1/2 pathway, and promotes cytotoxic effects through targeting p38 pathway (25). And (ii) multiple drugs to target different ERK and p38 or JNK pathways, such as ERK inhibitor (e.g., SOM230, OCT, or dopamine) plus p38 activator (e.g., cabergoline, bromocriptine, and fulvestrant) and/or JNK activator (e.g., UA). Also, fulvestrant can target both p38 and JNK pathways to promote the cell apoptosis for cell cytotoxic effects. These MAPK pathway-based combination therapies might produce better anti-cancer effects on PAs.

## Conclusion

This review summarized the studies of MAPK signaling in pituitary tumorigenesis. We discussed some important molecules involving in MAPK signaling pathway and potential drugs targeting the MAPK signaling (Figure 1 and Table 1). The ERK-MAPK signaling, p38-MAPK signaling, and JNK signaling all play important roles in PAs. Some therapeutic drugs exert anti-tumor effects by targeting one of these pathways or all these three pathways at the same time. MAPK signaling is a very complex network, and always interacts with other pathways such as PI3K and cAMP pathway to affect tumor progression. The latest development of MAPK signaling in PAs and the related anti-tumor drugs targeting MAPK signaling pathways would provide new insights on PA pathogenic mechanisms and pre-clinical data for treatment.

## Author Contributions

ML and YW collected and analyzed literature, prepared figures and tables, and wrote and revised manuscript. XZ conceived the concept, designed, coordinated, wrote and critically revised manuscript, and was responsible for its financial supports and the corresponding works. All authors approved the final manuscript.

## Conflict of Interest Statement

The authors declare that the research was conducted in the absence of any commercial or financial relationships that could be construed as a potential conflict of interest.

## Abbreviations

ACTH: Adrenocorticotropin
CIRP: Cold inducible RNA binding protein
CRH: Corticotropin-releasing hormone
CSAIDs: Cytokine-suppressive anti-inflammatory drugs
Eps8: Epidermal growth factor pathway substrate number 8
ERK: Extracellular signal-related kinase
GA: 18beta-glycyrrhetinic acid
GH: Growth hormone
GnRH: gonadotropin-releaseing hormone
GPCR: G protein-coupled receptor
IGF1: Insulin-like growth factor 1
JNK: Jun N-terminal kinase
LPS: Lipopolysaccharide
MAPKs: Mitogen-activated protein kinases
MKK: MAPK kinases
MKKK: MAPK kinase kinases
MKPs: MAPK phosphatases
MST4: Mammalian sterile-20-like kinase
PRL: Prolactin
RA: Retinoic acid
RKIP: Raf kinase inhibitory protein
RTK: Receptor tyrosine kinase
SAPKs: Stress-activated protein kinases
SSTs: Somatostatin analogs
SST: Somatostatin
TSH: Thyroid-stimulating hormone.
